# Population density and ranging behaviour of a generalist carnivore varies with human population

**DOI:** 10.1002/ece3.11404

**Published:** 2024-05-21

**Authors:** Brendan F. Alting, Benjamin J. Pitcher, Matthew W. Rees, José R. Ferrer‐Paris, Neil R. Jordan

**Affiliations:** ^1^ Centre for Ecosystem Science, School of Biological, Earth and Environmental Sciences University of New South Wales (UNSW) Sydney New South Wales Australia; ^2^ Taronga Institute of Science and Learning, Taronga Conservation Society Dubbo and Sydney New South Wales Australia; ^3^ Faculty of Science and Engineering, School of Natural Sciences Macquarie University Sydney New South Wales Australia; ^4^ Health and Biosecurity Department Commonwealth Science and Industrial Research Organisation Brisbane Queensland Australia

**Keywords:** anthropogenic resource supplements, camera‐trapping, carnivore, dingo, human wildlife conflict, SECR, urban ecology

## Abstract

Canid species are highly adaptable, including to urban and peri‐urban areas, where they can come into close contact with people. Understanding the mechanisms of wild canid population persistence in these areas is key to managing any negative impacts. The resource dispersion hypothesis predicts that animal density increases and home range size decreases as resource concentration increases, and may help to explain how canids are distributed in environments with an urban‐natural gradient. In Australia, dingoes have adapted to human presence, sometimes living in close proximity to towns. Using a targeted camera trap survey and spatial capture‐recapture models, we estimated spatial variation in the population density and detection rates of dingoes on Worimi Country in the Great Lakes region of the NSW coast. We tested whether dingo home range and population densities varied across a gradient of human population density, in a mixed‐use landscape including, urban, peri‐urban, and National Park environs. We found human population density to be a strong driver of dingo density (ranging from 0.025 to 0.433 dingoes/km^2^ across the natural‐urban gradient), and to have a negative effect on dingo home range size. The spatial scale parameter changed depending on survey period, being smaller in the peak tourism period, when human population increases in the area, than in adjacent survey periods, potentially indicating reduced home range size when additional resources are available. Our study highlights the potential value of managing anthropogenic resource availability to manage carnivore densities and potential risk of human‐carnivore interactions.

## Graphical abstract

We conducted a camera trap survey of dingoes in a heterogeneous landscape, and modelled their density across the region. Dingo density was highest in areas with highest human population density, and home ranges were smaller in these areas. This suggests dingoes are obtaining enough resources in areas with humans to have smaller home range sizes than in natural areas.

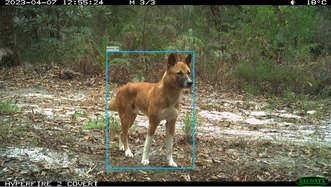

## 1 INTRODUCTION

Mammalian predators have adapted to urban and peri‐urban environments across the world, resulting in positive and negative interactions with people (Bateman & Fleming, 2012). To exploit the different types of resources available to them, individual animals utilising urban areas may exhibit behaviours distinct to their less urbanised counterparts (Klump et al., 2022). Understanding the population dynamics of carnivores is critical to managing their impacts in urban and peri‐urban environments. In particular, understanding how population density and territory size vary across a gradient from urban to natural areas is crucial to understanding the impact of anthropogenic factors on populations, which is key to informing effective management actions.

Successful urban adapted species often attain higher densities in urban areas compared to natural areas (Šálek et al., 2015), purportedly as the resources needed for survival are more highly concentrated in urban areas. Among carnivores, several canids thrive in urban environments. For example, the red fox (*Vulpes vulpes*) has adapted to cities globally, with densities up to four times greater in cities than in adjacent rural areas (Marks & Bloomfield, 1999), and human footprint is the biggest driver of fox home range size on a global scale (Main et al., 2020). Coyote (*Canis latrans*) populations are denser around clumped natural resources (Woodruff et al., 2021), and have also adapted to urban environments, where they can reach higher densities than in rural areas (Fedriani et al., 2001; Jonathan, 2011). Coyote home ranges are also smaller in urban environments (Grubbs & Krausman, 2009), indicating that they are obtaining sufficient resources from a smaller area than they would normally obtain from more natural settings. Elevated carnivore densities in urban environments can have significant flow‐on effects both for broader ecosystem functioning, such as increased competition with subordinate species (Wait et al., 2018), and through an increased likelihood of human‐carnivore interaction (Poessel et al., 2013). Understanding the drivers of elevated carnivore densities in urban areas can inform effective management; however, carnivore density estimates in urban environments are lacking in the literature, due to difficulty surveying in these areas and the cryptic nature of many carnivores.

The resource dispersion hypothesis describes group living in social species, suggesting that when resources are patchily distributed, group living will arise in a species as the costs of sharing space are reduced and outweighed by the benefits of accessing the resources within (Johnson et al., 2002). However, once the carrying capacity of the area is reached, further individuals are excluded. Territoriality describes this process, particularly the defence of part of a home range by an individual or group, to the exclusion of other conspecifics, and is near ubiquitous among carnivore species (Macdonald et al., 2019). With some exceptions (Pomilia et al., 2015), carnivore territory size and structure generally relate to resource availability, with larger territories observed in less productive environments (i.e. lower prey abundance; Duncan et al., 2015; Tallents et al., 2012). In landscapes with large quantities of a limiting resource, the area needed to support groups is reduced and may be so small as to make territorial defence unnecessary or unviable (Struller et al., 2022). Different sources of food supplementation are expected to affect animal populations in different ways. For example, Arctic fox (*Vulpes lagopus*) densities increase and territories disappear in the presence of a highly productive resource (Elmhagen et al., 2014). Black‐backed jackals (*Lupulella mesomelas*), however, reduce territory size around a clumped but highly productive food resource, but continue to maintain and defend territories around this resource (Jenner et al., 2011).

Humans can provide supplementary food resources to wildlife (hereafter anthropogenic resource supplements), either through direct feeding of wildlife (Takahata et al., 2023) or by allowing easy access to waste (Gort‐Esteve et al., 2024). These resource subsidies enable some animal species to attain high population densities in these areas (Bino et al., 2010). Urban areas and campgrounds provide interesting examples of consistent and intermittent resource subsidies, respectively. Urban areas are (usually) inhabited year‐round, and subsidies from here can provide consistent, predictable resources, allowing some carnivores to sustain high numbers throughout the year (Lowry et al., 2013). In urban areas then, according to the resource dispersion hypothesis, territoriality may be more difficult, or unnecessary, to maintain among groups, when food resources are consistently available and productive (Bino et al., 2010). In contrast, campgrounds are visited intermittently by people, mostly during summer months, holiday periods, and on weekends. It may be expected therefore that species with home ranges encompassing campgrounds will maintain territories, as the resource supplements that they utilise are likely intermittently delivered and cannot be consistently relied upon (Larson & Smith, 2019). We may expect therefore that territories in urban areas will be smaller than in less urbanised areas, even when the less urbanised areas are supplemented with an intermittent anthropogenic food resource.

Dingoes (variously *Canis dingo*, *C. familiaris*, and *C. lupus dingo*, among others) are Australia's largest terrestrial mammalian predator (10–18 kg; Smith et al., 2017) and have complicated relationships with humans. Previously present across the entire mainland, dingoes have since been extirpated from large areas in attempts to protect livestock (Cairns et al., 2020). Dingoes are socially monogamous, forming packs/family groups of 2–12 individuals, consisting of a breeding pair, pups, and young from previous years (Thomson et al., 1992). Dingoes, like other canids, typically defend territories, from which adjacent packs and transient individuals are excluded, although transient and dispersing individuals may temporarily co‐inhabit these areas (Thomson et al., 1992). Dingoes are generalist omnivores that are well adapted to exploiting anthropogenic resource supplements (McNeill et al., 2016; Newsome et al., 2013). The territorial nature of dingoes (Thomson et al., 1992), and their ability to consume a wide variety of food resources (Doherty et al., 2019), makes them a suitable model species from which to examine the relationship between anthropogenic resource supplements, population density, and ranging behaviours.

**FIGURE 1:**
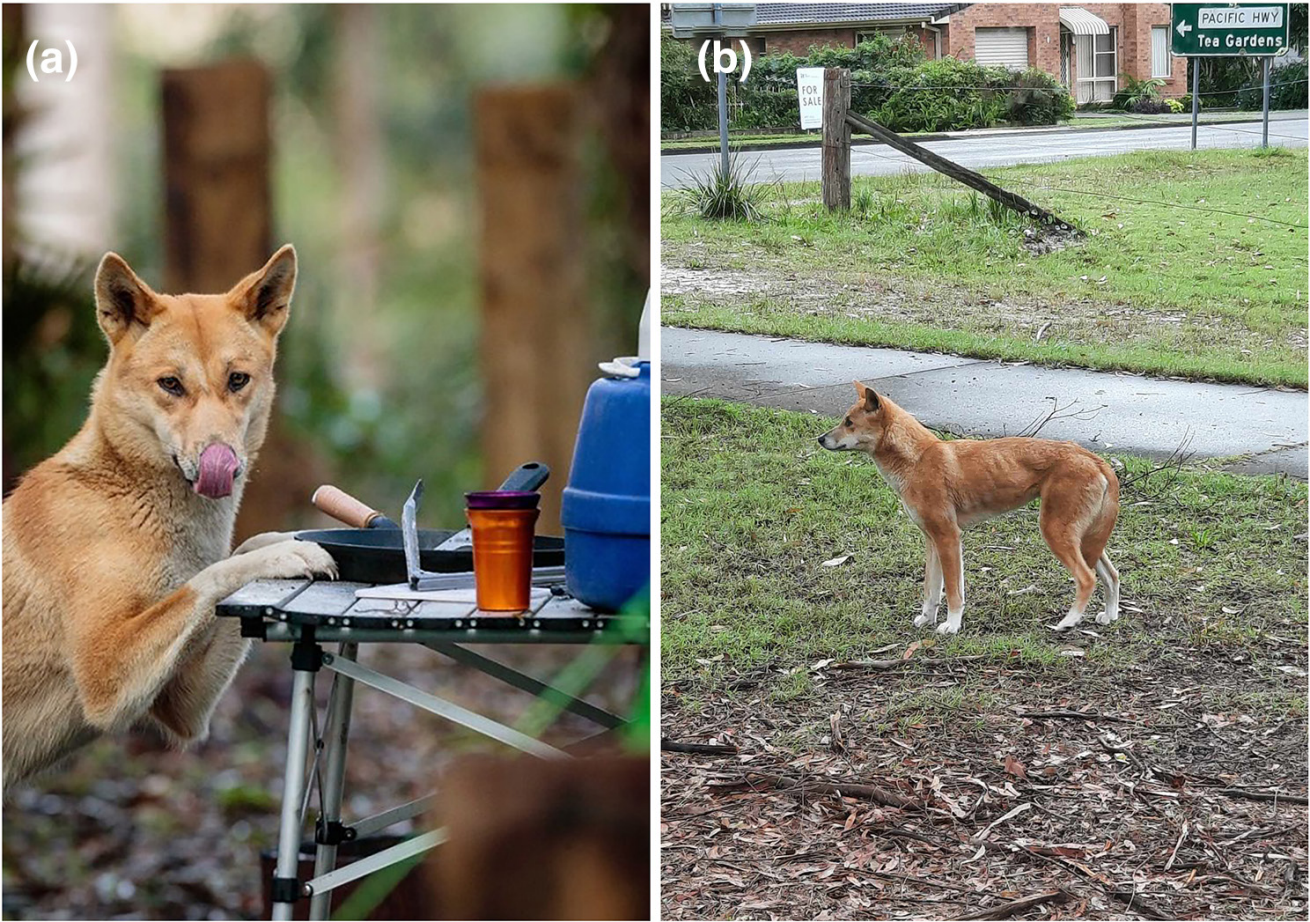
(a) Dingo attempting to steal food from a campground, and (b) dingo in the town of Hawks nest, both in the Great Lakes region of New South Wales, Australia, 2021–2023. Photo credits B‐J Vial (a) and B Alting (b).

Dingoes are known to change their behaviour in response to resource supplementation, becoming bolder and more accustomed to human presence (Figure 1), such as in campgrounds (Behrendorff, 2021). When in close contact with humans, dingoes can pose management challenges, despite their cultural importance for much of the population (O'Neill et al., 2017). For example, if dingoes become habituated to people, conflict can occur as dingoes can become aggressive when expecting food or encountering domestic pets. These issues can arise in places such as peri‐urban settings (Hine et al., 2020) and campgrounds (Thompson et al., 2003), and individual dingoes or their populations may be lethally controlled in attempts to avoid any or further conflict (Behrendorff, 2021). Occasional physical interactions with humans do occur, almost always in areas in which dingoes have been fed by humans, or have stolen food from them, and consequently become accustomed to human presence (Burns & Howard, 2003). While dingo behavioural changes around anthropogenic resource supplements are well observed (Newsome et al., 2013), less well known is whether dingo population density and home range size change with human population density.

The influence of the resource dispersion hypothesis on dingo population structure has mixed support. Newsome et al. (2013) showed that dingoes close to an anthropogenic resource supplement at a mine site had smaller home ranges than those further away. While the mine study used data from GPS collars, a more recent study from far north Australia using camera traps showed no clear effect of anthropogenic resource supplements (in this case a rubbish disposal site) on density or home range size (Gabriele‐Rivet et al., 2020). Dingo detectability in the north Australian study was also lower at camera stations situated at the resource supplement than on trails. Population estimates for dingoes are lacking in the literature, primarily due to a lack of research focus, and relative measures of abundance are often used to infer population size (Allen et al., 2011; Wallach et al., 2009). This is concerning because relative abundance indices can be biased by changes in behaviour and detectability (Witmer, 2005), potentially limiting their use in predator management and decision‐making.

Population models have been developed which disentangle behavioural and numerical processes to enable reliable estimates of animal density. Spatial Capture‐Recapture (SCR) models are considered the gold standard of population estimates (Efford & Fewster, 2013; Royle et al., 2013). This class of models has been used to estimate dingo abundance in some regions in Australia. Gabriele‐Rivet et al. (2020) used spatial mark resight models, a type of SCR model in far northern Australia, and estimated dingo density of 0.15 (95% CI: 0.14–0.16) dingoes/km^2^ in their study area (606 km^2^) in the summer months. Forsyth et al. (2019) used SCR models in Namadgi National Park (298 km^2^), in South‐East Australia, and estimated a density of 0.06 (95% CI: 0.03–0.11) dingoes/km^2^, almost half that of populations in northern Australia. Robley et al. (2018) using SCR models estimated dingo density at 0.03 dingoes/km^2^ (95% CI: 0.02–0.03) over a much larger study area in South‐East Australia (1844 km^2^). SCR models require long‐term, large‐scale deployments of traps/camera traps, accurate identification of individuals, and complex statistical analysis. The combination of these requirements means these studies take time and can be prohibitive to management practitioners. Despite nearly 200 years of trying to manage dingoes, we currently have little understanding of how dingo density changes between and within regions, particularly around peri‐urban areas.

In this study, we assessed dingo population parameters and their possible relationship with human population density in and around Myall Lakes National Park, on Worimi Country in the midcoast of New South Wales, Australia. We used a grid of remote cameras spanning a gradient of urban influence and developed SCR models to estimate dingo density across the study area. We predicted that higher human population density would be associated with (1) a higher density of dingoes, (2) smaller home‐range sizes, and (3) higher detection rates of dingoes, due to reduced home‐range size and behavioural changes therein. We collected data and tested these predictions across 2 years during periods of high human occupancy at campgrounds, caravan parks, and urban areas. We also provide the first estimates of dingo density in a mixed use temperate coastal ecosystem in eastern Australia.

## 2 METHODS

### 2.1 Study site

**FIGURE 2:**
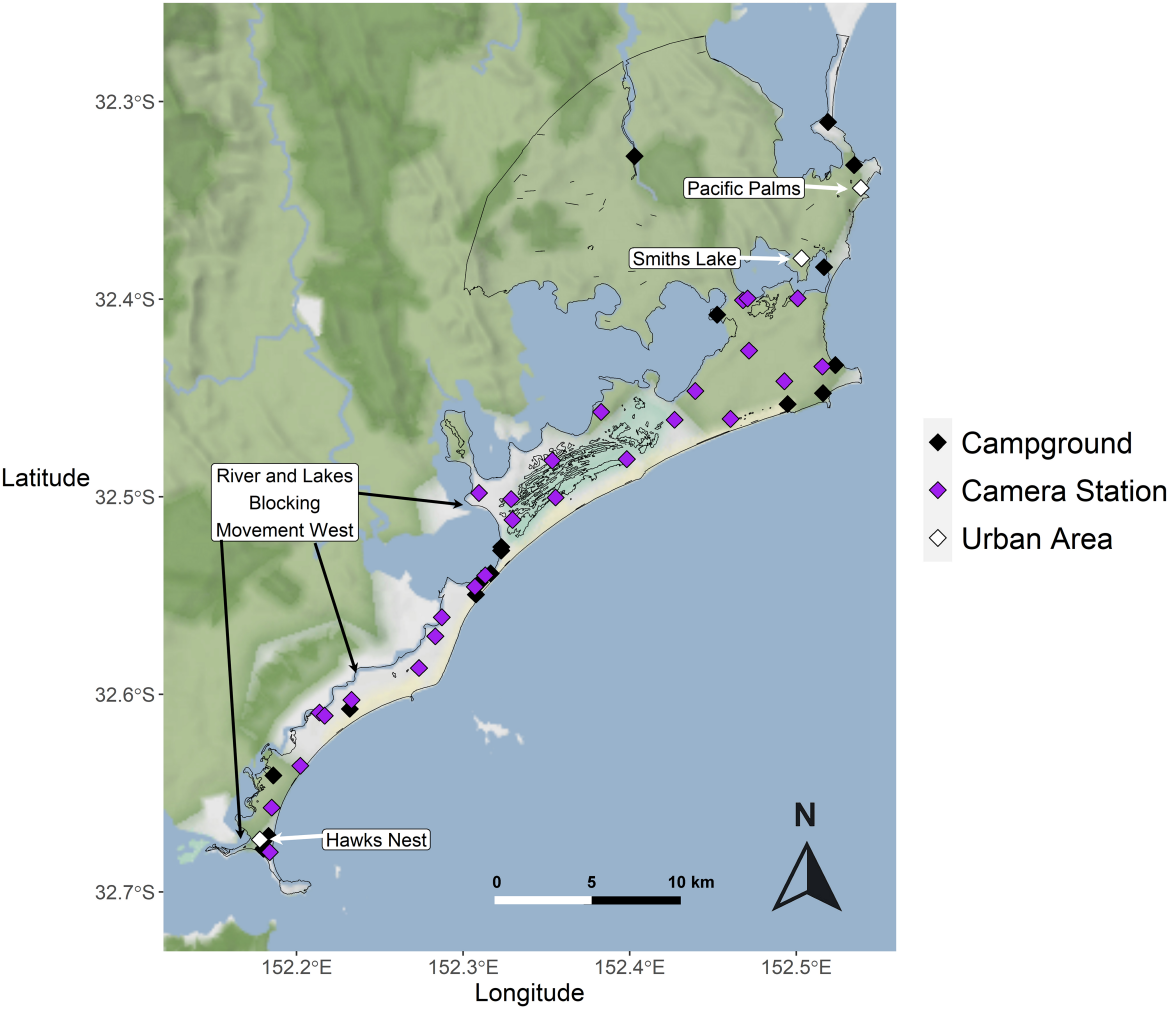
The Great Lakes region on Worimi Country on the mid‐coast of New South Wales, Australia. Light outline represents habitat mask used for dingo capture‐recapture population modelling. Purple dots = Camera Trap Stations. Black dots = Campgrounds. White dots = Urban Areas. Figure made in Rstudio using ggplot2 and ggmap (Kahle & Wickham 2013; Wickham 2016). Camera stations are spaced on average 2380 m apart, some appear close together as they were moved between sessions due to theft.

The Great Lakes region is a mixed‐use coastal area situated on the mid‐coast of New South Wales, Australia (centred on 32.492° S, 152.343° E). The majority of the study area is part of Myall Lakes National Park, although there are multiple areas of human habitation outside the park including the ‘key housing localities’ of Hawks Nest (permanent population 1413), Smiths Lake (permanent population 1332), and Pacific Palms (permanent population 668; MCC Housing Strategy Plan 2023; Figure 2). The area is used for recreation such as fishing, camping, boating, and bike riding in the national park and towns. The area is enclosed to the west and south by Lakes, and to the east by the ocean. The main point of connectivity to the area is through the northern connection to adjacent national parks, farmland, and towns. A road bridge also connects Hawks Nest to Tea Gardens, and is sometimes, but rarely, crossed by dingoes. The study area consists of numerous vegetation types, including sand dunes, open and closed forest dominated by *Angophora costata* and *Banksia serrata*, coastal wetlands, littoral rainforests, and paperbark swamps dominated by *Melaleuca quinquenervia* and *Eucalyptus robusta*. Twenty campgrounds/caravan parks are found throughout the study site, including in urban areas, ranging from 3 to 200 campsites in each campground. Indiscriminate lethal control of dingoes through trapping and poison baiting is not practised in the study area, but dingoes are sometimes controlled when their behaviour around people is deemed a risk to public safety (MCC Dingo Management Plan 2019).

### 2.2 Camera trap deployment and image processing

A camera trap survey was conducted across the same site in two consecutive years. Twenty‐two paired camera stations (*n* = 44 cameras; Reconyx Hyperfire H2x1000) were deployed for 181 days between December 2021 and June 2022 (Table 1). Twenty‐one paired camera stations (42 cameras) and one single camera station were deployed for 179 days between December 2022 and June 2023 (Figure 2). A random square grid, with grid side lengths of 2 km, was generated in Arcmap (v10.8, ESRI 2011) and overlaid onto the study area. Camera stations were then placed at the centre of every second grid square, diagonal to the previous grid square with a camera station within it (due to the irregular, northeast orientation of the study area). Camera stations were then moved to the nearest trail or road from that centre point. This resulted in camera stations being spaced on average 2380 m apart across the different sessions (range: 2189–2420 m). Dingoes in a peri‐urban setting in Queensland had a mean home range of 17.7 km^2^ (Allen et al., 2013) and so our camera trap spacing meant individuals could be detected in multiple parts of their home range (Efford & Fewster, 2013). Camera stations consisting of two cameras, one on either side of a trail, were set to take three photos per trigger, with no interval between triggers (rapidfire) and medium‐high sensitivity, and secured with python locks 50–100 cm high on trees (Meek et al., 2014). Paired cameras ensured that both flanks of each individual dingo were captured. Cameras were serviced on average every 36 days (range: 28–45 days) to replace batteries and SD cards. Cameras were active for a total of 7646 camera trap nights. The majority of camera stations remained in the same location throughout the survey, although some camera stations were moved/replaced after being stolen or when environmental conditions such as floods or vegetation growth rendered cameras non‐operational. This variation in camera effort was accounted for in the modelling by considering cameras as ‘non‐operational’ when stolen or covered by vegetation.

**TABLE 1:** Summaries of session durations and survey periods for a dingo camera trap survey conducted on Worimi Country in the Great Lakes region on the mid‐coast of New South Wales, Australia.

Session (survey period)	First date	Last date	Survey length (days)	Total camera stations
1 (A)	02/12/2021	31/01/2022	60	22
2 (B)	01/02/2022	31/03/2022	60	24
3 (C)	01/04/2022	31/05/2022	61	22
4 (A)	05/12/2022	01/02/2023	59	21
5 (B)	02/02/2023	01/04/2023	59	22
6 (C)	02/04/2023	01/06/2023	61	22

*Note*: Survey period refers to what time of year each session was conducted in both years (A = December–January; B = February–March; C = April–May).

Camera trap images were sorted into folders and timestamped in R (v4.3.1, R Core team, 2023), using camtrapR (Niedballa et al., 2016). After images were tagged with dates and camera trap station, we used megadetector v3.0 (Microsoft, 2023), a machine learning program which classifies each image as containing an animal, vehicle, human, or none of the above, and has been shown to be highly effective at detecting mammals in particular (Beery et al., 2019). Using the Timelapse image processing software (Greenberg, 2020; Greenberg & Godin, 2015), we set a minimum threshold of 15% confidence of an animal being in the image, and manually sorted all ‘animal’ images, tagging each image with the species present. Once images were classified to species level, we then separated images containing dingoes for further analysis.

### 2.3 Dingo identification

**FIGURE 3:**
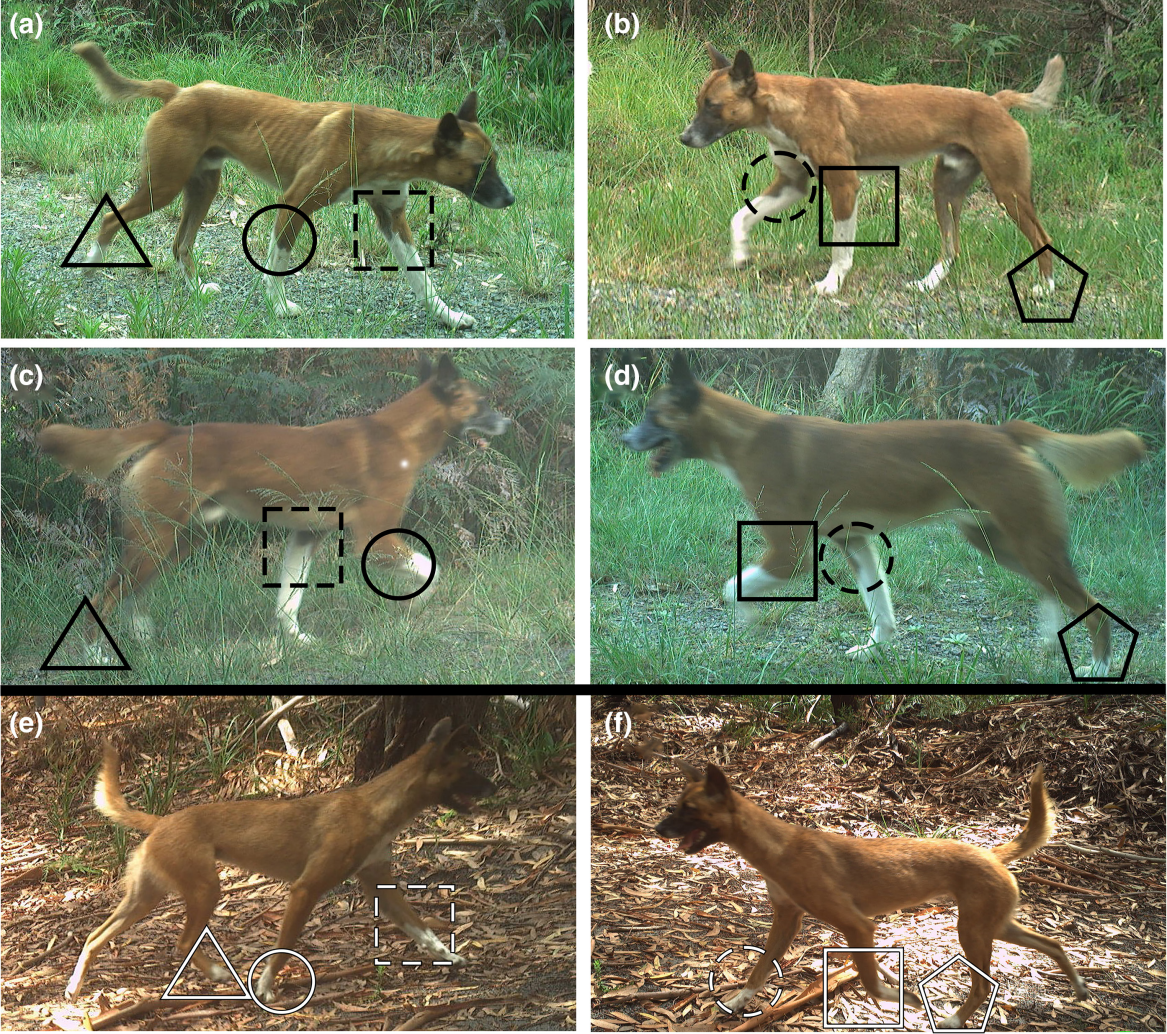
Two dingo identity profiles identified from a camera trapping survey in the Great Lakes area of New South Wales, Australia, from 2021 to 2023. Left and Right panels for each row are images taken at the same time of each dingo, with camera traps on either side of a trail facing inwards. (a, b) UOM1707 in 2021. (c, d) UOM1707 in 2023. (e, f) UOF2009 in 2021. Identifying features are the unique ‘sock’ patterns, highlighted by the shapes (square = front left leg, circle = front right leg, pentagon = back left leg, triangle = back right leg). Dashed shapes are inside leg features, solid shapes are outside leg features. Note the higher position and curved shape of the white ‘socks’ on the front legs of UOM1707, both inside and outside, compared to UOF2009.

Dingoes were independently identified to the individual level by two researchers. A dingo identity profile was created each time a new individual was detected on the cameras, consisting, where possible, of clear images of the left and right flank (Figure 3). Dingoes were identified using their sock patterns, which differ in length and shape and are unique to each individual dingo in the study area. We also used scars, muzzle colour, and coat colour (Gabriele‐Rivet et al., 2020) as further identifying features. GPS collars were also present on six dingoes in the area as part of the Myall Lakes Dingo/Dapin Project, which aided in identification of some individuals, along with reference to their existing identification database. Collectively, these features were sufficient to identify all clear daytime dingo images recorded on camera traps, and thus the entire population was available for sampling. The two researchers then compared their identifications and discussed any differences in opinion, which were resolved after discussion. If consensus could not be reached, a third researcher would adjudicate. We could not reliably identify individuals at night with infrared flash photos, and so we discarded all nocturnal images for SCR analysis (Table 2). Daytime images containing unidentifiable dingoes, due to blurry photos or only part of the animal in the image, were also discarded for SCR analysis. This may result in underestimates of dingo density and detectability, particularly if there were individuals which preferred nocturnal movements. However, based on prior GPS telemetry of this dingo population (MLDP, unpublished data) individuals were active throughout the diel cycle and so are unlikely to be completely undetected in daytime across the survey periods. Pups (dingoes <1 yo) were differentiated from adults by their considerably smaller size, particularly in the earlier periods of the survey.

**TABLE 2:** Number of adult dingo individuals identified, total number of dingo detections (day and night), and unidentifiable daytime images (from blurry photos) for a dingo camera trapping survey from the Great Lakes region of New South Wales, Australia.

Session	No. of adult individuals detected	Adult daytime dingo detections	Unidentifiable daytime detections	Nighttime dingo detections	Movement recaptures	Adult dingoes/km^2^ (+/− 95% CI)
1	24	266	5	294	164	0.093 (0.059–0.146)
2	22	219	9	193	138	0.076 (0.047–0.122)
3	18	198	18	312	128	0.058 (0.035–0.096)
4	17	150	5	151	80	0.067 (0.040–0.112)
5	22	166	9	301	93	0.077 (0.048–0.122)
6	17	188	3	317	119	0.055 (0.033–0.092)

*Note*: Density estimates from spatial capture‐recapture modelling of the population.

Pup attrition in canids in the period from leaving the den until adulthood can be as high as 50% (McNutt & Silk, 2008; Sidorovich et al., 2017). Pups were therefore excluded from SCR analysis to reduce the likelihood of violating the assumption of nil mortality in SCR models, and because the dependence of dingo pups on adults may result in different movement behaviours to adults, particularly in their first few months out of the den. Consistent with other studies, we considered detection events of the same individual to be independent when detections occurred >30 min apart (Gabriele‐Rivet et al., 2020).

### 2.4 Spatial kernel density estimates of human population density

We calculated indices of human population density using kernel density estimates. Areas of human occupation were considered to be either large campgrounds or caravan parks within the study area (*n* = 17) and the urban areas described above (*n* = 3; Figure 2). Smaller campgrounds in the area (<10 campsites) were excluded as they were considered too infrequently occupied to provide a significant resource. We created a weight metric for each area to reflect the different human population density in each area. For urban areas, we used the number of permanent dwellings as the weight for each area (ABS 2021; MidCoast Council, 2023). For campgrounds and caravan parks, we used the number of available camping sites (NSW NPWS 2024). We used the same kernel density estimates of human population for each survey period, as while the numbers of people in the area fluctuate over time, it is difficult to quantify by how much in each session, and the off‐peak populations are likely proportionally similar.

Kernel density estimates of human population size were calculated using the ‘kde’ function in the spatialKde package (Caha, 2023), using the assigned weights for each centre of human population. We used a grid cell size of 500 m and a bandwidth of 5000 m to estimate kernel density and then log transformed these values to use as our human population density index.

### 2.5 Spatial capture‐recapture modelling of dingo population

SCR models require a ‘habitat mask’ of the study area, within which any individual animals could potentially be detected by the ‘trap’ array (Figure 2). This mask may contain spatial covariates, to model spatial variation in density. A shapefile of the study area was created in ArcMap V10.6 (2023). We used a buffer of 14 km around camera‐trap stations to ensure we estimated density across an area where all individuals could have been exposed to camera‐trap stations (Royle et al., 2013). This dingo population has been studied since 2018, and telemetry data from collared dingoes in the area indicates that resident dingoes tracked during the camera deployment period did not leave the area captured by the 14 km buffer (MLDP, unpublished data). We then created a habitat mask (6301 pixels) using this shapefile and the SECR package in R (Efford, 2024). This resulted in a total habitat mask area of 347 km^2^. Each pixel in the habitat mask was 0.055 km^2^. This fine scale resolution relative to dingo home range size was necessary in our study to ensure that small peninsulas and irregular land shapes were included in the mask, and not discarded as non‐habitat.

**FIGURE A1:**
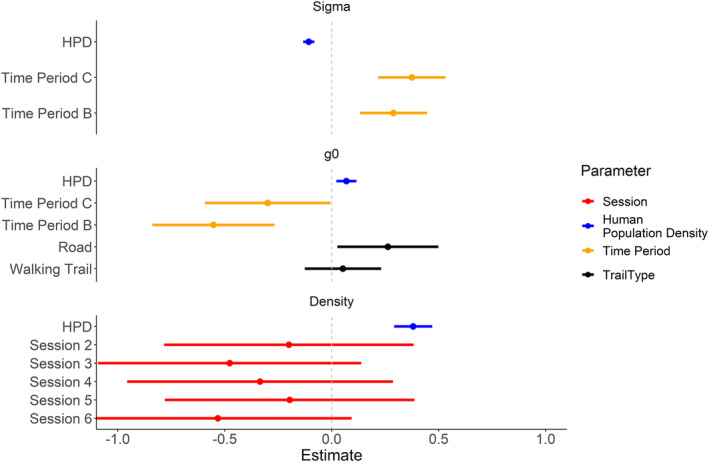
Kernel density estimates (KDE) of human population size in the Great Lakes, New South Wales, Australia. KDE weights were taken from number of houses in urban areas, and number of campsites in campgrounds and caravan parks. This was used as a covariate in modelling dingo population density across the study region, using spatially explicit capture recapture models, in R using the ‘secr’ (Efford, 2024).

We assigned the kernel density estimates of human population density to each pixel in the habitat mask, using the overlap package in R (Ridout & Linkie, 2009) (Figure A1). When estimating *σ* and *g*_0_ using covariates for models in SECR, covariates must be assigned to each camera station specifically. We overlaid all camera trap locations onto the habitat mask, extracting the corresponding pixel for each trap location. We then took the human population density estimate value from this pixel and assigned it to each camera trap station as a covariate.

We excluded all Freshwater Wetlands (NSW STVM vegetation forms; NSW state gov, 2022) from the habitat mask as GPS collar data shows dingoes in this region very rarely enter this habitat (MLDP, unpublished data). The southern border of the study area forms a natural peninsula bounded by a river to the west, but a road bridge now connects Hawks Nest to the town of Tea Gardens across the water. We chose to exclude habitat on the west side of this bridge from Hawks Nest, as while dingoes are known to cross this bridge (MLDP, unpublished data), these events are rare, and movement across the river is almost completely restricted. Additionally, lethal control of dingoes is far more common on the other side of this bridge as the surrounding areas include farmland, and including these areas would potentially violate the assumptions of no deaths or emigrations from the study area.

SCR models use spatial information from the locations of the traps to estimate the activity centre of the animals and derive estimates of animal density (Green et al., 2020), particularly improving reliability of estimates for cryptic, low density and wide‐ranging carnivore species compared to non‐spatial approaches (Harmsen et al., 2020). SCR models link a separate detection and process model to estimate population density, as well as two detectability parameters: *g*_0_ and *σ*. The detection probability (*g*_0_) of an individual animal is its likelihood of being detected if a camera station is positioned at the animal's hypothetical activity centre. The spatial scale parameter, sigma (*σ*), is a measure related to home range and is directly proportional to home range size (Petit et al., 2018). Together, these detectability parameters calculate the likelihood of an individual being detected at a camera station.

We broke up our continuous camera‐trap monitoring into six discrete ‘sessions’ of approximately 60 days each (Table 1), to reduce the likelihood of deaths or emigrations from the population during each session. The different sessions for capture history of the dingoes were assigned covariates with three levels, ‘A’ (sessions 1 and 4, December & January), ‘B’ (sessions 2 and 5, February–March), and ‘C’ (sessions 3 and 6, April–May), as they spanned the same period of the year in the two years of study (Table 1).

We ran multi‐session, closed population SCR models in R in the ‘SECR’ package (v 4.6.1, Efford, 2024) with an exponential detection function. We expected that all dingoes in the area were available for sampling, due to the distinct pelage patterns of each individual, and that dingoes previously GPS tracked in the area were active for significant parts of the day (MLDP, unpublished data). We used a closed population SCR model which assumes no deaths or emigrations of individuals from the study area, although violations of this assumption are inevitable in wild ecosystems. Our study population is more likely to be closed than others, given it is predominantly only accessible from one direction (north), with very rare crossing of a human‐dominated bridge in the south. Migration by dingoes to the west of the study area is rare, due to geographic features such as the Myall Lake in the central west of the study area, and dingoes must go north to go around this lake to disperse to, or emigrate from, the west (Figure 2).

We first fit a null model, with all parameters held constant, with no spatial or temporal variation in model parameters. We then fit a full model, which estimated density separately in each session, and where density, *σ* and *g*_0_ varied as a function of human population density, *σ* and *g*_0_ varied as a function of ‘Survey Period’, and *g*_0_ as a function of ‘Trail Type’ (four wheel drive track, unsealed road, walking trail; Table 2). We included these covariates as carnivores can be more or less detectable on different types of trails, as they may be more likely to use a well‐travelled road, such as a 4wd track, rather than a walking trail (Chutipong et al., 2014). We chose to vary density by session, rather than by survey period. Dingo population size may vary through time, due to stochastic population changes such as territorial conflicts or road accidents resulting in death, or emigration from an area. As dingoes are resident and hold territories year‐round, density is not expected to alter significantly depending on the time of year. *σ* and *g*_0_, however, may potentially change depending on the time of year, as behaviours may change due to resource availability or biological effects such as the breeding season (Cronk & Pillay, 2021). Alternative models with combinations of constant and non‐constant components were also tested.

We then compared all models using AICc and selected the top ranked model. We assessed statistical evidence for model parameters based on confidence interval overlap of the coefficient estimates, and a *p*‐value threshold of .05. A *p*‐value below .05 is reached once the 95% confidence intervals of the coefficient estimates do not overlap 0 on the logarithmic scale.

## 3 RESULTS

A total of 854,187 images were captured across all survey periods. After megadetector processing, 160,486 ‘animal’ images remained (although due to the low confidence threshold we set, some ‘false positive’ images remained and did not necessarily contain an animal). Of these, we categorised 35,029 images as containing a dingo. 22,147 of these images were either at night or were too blurry to identify dingoes from. The remaining 12,884 images consisted of 1187 individually identifiable adult dingo detection events across the survey period, and 49 unidentifiable daytime images (Table 2). Thirty‐three adult dingoes were identified in total across the 2 years ($\bar{x}$ = 21.5; SD ± 2.7). We identified 27 pups across the 2 years: 14 in the first year, and 13 in the second year. Of the adult individuals identified in the second year (sessions four, five, and six), eight were identified as pups in the previous year (sessions one, two, and three), and were only used in modelling in the second year. We obtained between 150 and 266 independent adult dingo detection events for each session (Table 2).

**FIGURE 4:**
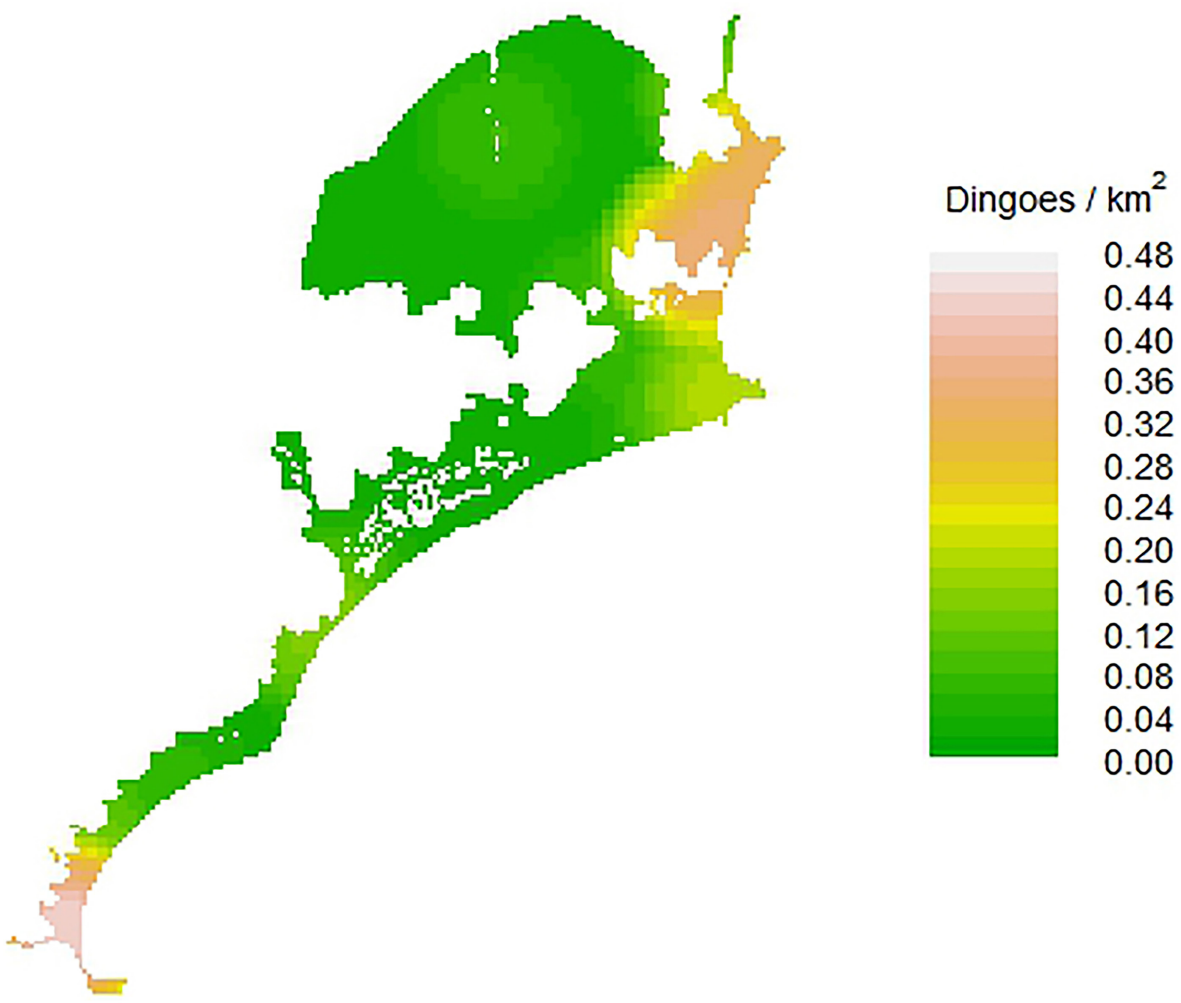
Example dingo density surface estimates (Dingoes/km^2^), varying by human population density in the Great Lakes Region, NSW, Australia. Density surface from session 1 (Dec 2021–Feb 2022) is presented here as the density surfaces from all sessions illustrate the same trend. Note higher density in the urban area in the towns in the south and north of the study area, and slightly higher density near campgrounds.

**FIGURE 5:**
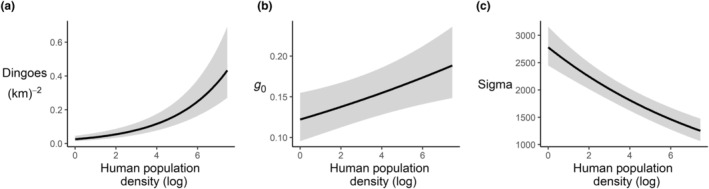
Marginal effects plot of human population density (95% CIs) on model parameters for a SCR model of dingoes in the Great Lakes Region of New South Wales, Australia (2021–2023). (a) Density, (b) Detectability (*g*_0_), (c) Sigma (*σ*). All effect values from model parameter are predicted at a reference level of session = 1, track type = 4wd track, and Survey Period = A.

The model containing covariates had significantly more support than the next highest model (dAICc = 13.29; Table 3). In the top ranked model, there was a significant positive effect of human population density on dingo density (estimate = 0.381 (95% CI: 0.292 to 0.470); Figures 4 and 5a). There was also a significant positive effect of human population density on *g*_0_ (estimate: 0.069, 95% CI: 0.022 to 0.116; Figure 5b) and a negative effect of human population density on *σ* (estimate = −0.107, 95% CI: −0.133 to −0.081; Figure 5c).

**TABLE 3:** SCR models using camera trap data from a dingo survey on the mid‐coast of NSW, Australia with AICc values.

Model	*D*	*σ*	*g*_0_	AICc	dAICc
Full	~Session + HPD	~Survey Period + HPD	~Survey Period + HPD + Trail Type	10,643.72	0
*D* and *σ*	~Session + HPD	~Survey Period + HPD	~1	10,657.01	13.29
*σ* and *g*_0_	~1	~Survey Period + HPD	~Survey Period + HPD + Trail Type	10,712.85	69.13
*D* and *g*_0_	~Session + HPD	~1	~Survey Period + HPD + Trail Type	10,720.95	77.23
Only *σ*	~1	~Survey Period + HPD	~1	10,729.86	86.14
Only *D*	~Session + HPD	~1	~1	10,732.66	88.93
Only *g*_0_	~1	~1	~Survey Period + HPD + Trail Type	10,769.60	125.88
Null	~1	~1	~1	10,775.90	132.18

*Note*: ‘Full’ model had the most support. *Sessions*: Session 1 = December 2021–January 2022–60 days; Session 2 = February 2022–March 2022–60 days; Session 3 = April 2022–May 022–61 days; Session 4 = December 2022–February 2023–59 days, Session 5 = February 2023–March 2023–59 days, Session 6 = April 2023–May 2023–61 days. *Survey Period*: ‘A’ = December–January, ‘B’= February–March, ‘C’ = April–May. HPD = Kernel density estimates of human population density across study area.

**FIGURE 6:**
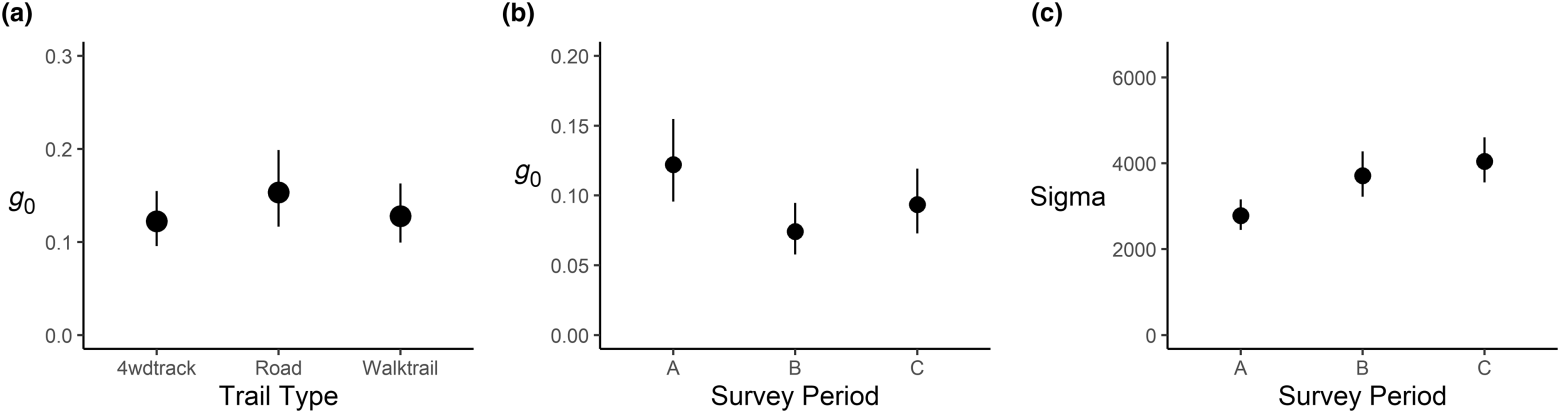
(a) Conditional effect plot of session on dingo detectability (*g*_0_), using reference levels of human population density = 0, session = 1, Survey Period = ‘A’, from a camera trap survey in the Great Lakes region of New South Wales, Australia. (b) Conditional effect of Survey Period on model parameters detectability (*g*_0_) and (c) (*σ*) using reference levels of human population density = 0, session = 1 and trail type = 4wd track. Survey period A: December–January, B: February–March, C: April–May.

**FIGURE A2:**
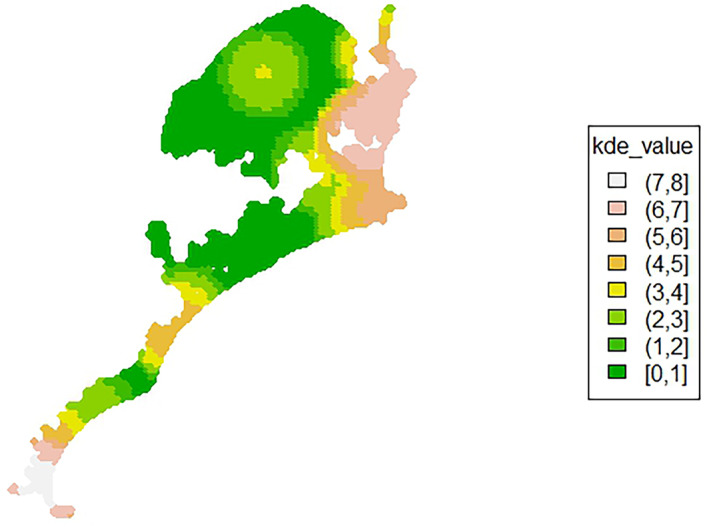
Model coefficient estimates and associated 95% confidence intervals for a spatial capture recapture camera trapping study of dingoes in the Great Lakes New South Wales, Australia. Categorical variables Time Period, Trail Type, and Session are calculated by comparing them to reference levels (Time Period: A, Trail Type: 4wd Track, Session: 1). We identified statistical significance for each parameter if 95% CIs did not overlap zero. The model parameters we found statistical evidence for were human population density on dingo density D (+), sigma *σ* (−) and detectability *g*_0_ (+), Time Period C and B (relative to A) on detectability *g*_0_ (−), and on sigma *σ* (+), and Trail type ‘Road’ on *g*_0_ (+) (relative to 4wd track).

We found no statistical evidence that adult dingo density varied across the six sessions (Figure A2). Density was estimated to range from 0.055 (95% CI: 0.033 to 0.092) to 0.093 dingoes/km^2^ (95% CI: 0.059 to 0.146) across the six sessions, with a $\bar{x}$ of 0.071 dingoes/km^2^ (95% CI: 0.044 to 0.115; Table 2). Dingoes were significantly more detectable (*g*_0_) on unsealed roads than 4wd tracks or walking trails (estimate = 0.263, 95% CI: 0.027 to 0.499; Figure 6a). *g*_0_ was significantly lower in survey period ‘B’ (estimate = −0.553, 95% CI = −0.838 to −0.267) and survey period ‘C’ (estimate = −0.300, 95% CI = −0.593 to −0.007) than in survey period ‘A’ (Figure 6b). *σ* was significantly higher in survey period ‘C’ (estimate = 0.133, 95% CI = 0.132 to 0.445) and survey period ‘B’ (estimate = 0.375, 95% CI = 0.217 to 0.532) than in survey period ‘A’ (Figure 6c).

## 4 DISCUSSION

Human population density had a positive effect on dingo density estimates, with dingo density being highest in areas with a high human population, such as the town of Hawks Nest in the south of the study area (Figure 4). This agrees with other studies that have shown that the density of some generalist carnivores can increase near human activity (Bateman & Fleming, 2012). Additionally, we found an effect of human population density on *σ* (Figure 5c), suggesting that the dingo home range was smaller around human habitation, as the home range scales with the spatial scale parameter *σ* (Petit et al., 2018). That dingo density increased with increasing human population density indicates that conditions in these peri‐urban environments are more productive for dingoes than in more ‘natural’ areas. One reason that has been proposed for this is an increase in food resources available in urban areas, as generalist carnivores have been shown to readily exploit anthropogenic food supplements (Bateman & Fleming, 2012). Indeed, dingoes have been shown in some instances to obtain higher densities and have smaller home ranges around anthropogenic food sources, such as waste dumps (Newsome et al., 2013), and are often highly visible around campgrounds (Behrendorff, 2021), and in our study area are often observed taking food from campgrounds and urban areas (Figure 1).

Animal density has been shown to increase in some instances when geographical features, such as fences, restrict animal movements (Riley et al., 2006) referred to as ‘home range pile up’. This has been illustrated in carnivores such as bobcats, that increased in density around urban landscape features that impeded movement (Schmidt et al., 2023). Hawks Nest is geographically isolated by water to the south, west, and east, and only one long and exposed road bridge connecting it to land further west, potentially resulting in dingo ‘home range pile up’ in this area (Figure 2). This phenomenon, combined with anthropogenic resource supplements available in urban areas, may help to explain why dingo density was highest around the large town of Hawks Nest in the south of the study area. However, the positive effects of geographic barriers on population density are predicted to decline over time (Krebs et al., 1969), and other studies have found no support for the home range pile‐up hypothesis (Lewis et al., 2015). We consider it more likely that anthropogenic resource supplements are driving population density in dingoes in this region, rather than home range pile up, given that high dingo density was also observed in areas with geographic connectivity to the north of the study area near Smiths Lake where there are no geographic barriers to movement (Figure 4).

While the observed effect of human population density on *σ* was significant, this effect was smaller than predicted. It is possible that our camera trap deployment, with only 20 cameras, did not identify and recapture dingoes on sufficient cameras to identify a stronger trend. Supplementary evidence supports this in that one individual dingo observed by researchers in person from the study area in 2021 was never detected on the camera station present in Hawks Nest despite being seen nearly daily in Hawks Nest, often in close proximity to humans and within 1 km of the camera station. Hawks Nest is the most densely populated urban location in our study area, and potential resource subsidies derived from humans would be expected to scale with population size. Indeed, the resident dingoes identified in Hawks Nest, the area with the highest potential resource subsidies, were nearly always seen only at the camera station in Hawks Nest, and never further north than one camera station 2 km outside of town, indicating a very small home range for these individuals. It is possible therefore that the home ranges of individuals in high human population density areas may be too small to be detected on camera stations spaced 2 km apart (Figure 2). Our camera stations were equally spaced, as generally recommended for pilot camera trapping studies (Royle et al., 2013) where knowledge of the broader population is limited. Given the flexibility of SCR survey designs with regard to trap placement (Murphy et al., 2019), future camera trapping studies may place more camera trap stations around high human population areas to obtain more captures of individuals in these locations.

### 4.1 Session/seasonal fluctuations

For carnivores, home range sizes can fluctuate considerably depending on season (Brandell et al., 2021; Comley et al., 2023). Our estimates of the spatial scale parameter *σ* varied depending on survey period, with *σ* being higher in survey period ‘C’, compared to survey periods ‘A’ and ‘B’ (Figure 6c). This may be because during the initial 60 day survey period ‘A’, human campground and urban areas are fully occupied due to the holiday period, and this may provide ample food resources for dingoes, potentially reducing the distances needed for dingoes to travel (Newsome et al., 2013). In the survey periods, ‘B’ and ‘C’, this transient human population decreases in the area, and dingoes may reduce in density around focal resources, or need to travel further to obtain their resources than during survey period ‘A,’ the peak summer holiday period. Another reason for the observed change in *σ* could be that the dingo pre‐breeding period occurs from February to April, and the breeding period from April to June, and these periods may coincide with increased movement distances as transient individuals search for new territories, as occurs in coyotes (Sasmal et al., 2018). *g*_0_ also decreased in survey periods ‘B’ and ‘C’ (Figure 6b). This is to be expected, as *g*_0_ is usually negatively correlated with the *σ* parameter (Royle et al., 2013). Individuals with a larger home range are less likely to be detected at a location at a given time, compared to individuals with a smaller home range (Gorosito et al., 2022). Whether the effects of anthropogenic resource supplementation or changes in the dingo breeding season are driving the changed ranging patterns of dingoes requires further study.

At a finer scale, dingo density did not change significantly between sessions, despite considerable interpack conflict occurring during the second and third sessions. Six dingoes were known to have either died from interspecific conflict (three) or dispersed beyond the study area (three) between February 2022 and April 2022, potentially violating the assumptions of no deaths or emigrations from the study area, had camera traps only been active for one session that contained these events. Dingo density did not vary significantly between sessions, but was highest in session one (0.093 dingoes/km^2^), declined in session two (0.076 dingoes/km^2^) and declined again in session three (0.058 dingoes/km^2^; Table 2). That the model suggested a small decline in dingoes that was conjointly observed in the field indicates that our SCR models performed well and could detect changes in population size. Indeed this study provides an important validation of the model, highlighting the importance of conducting surveys in multiple sessions. SCR models assume no deaths or emigrations from the population, but in reality animal deaths are inevitable in wild populations, and these deaths will often go undetected in camera trap surveys. This issue highlights the benefits of conducting capture‐recapture surveys across multiple seasons and years.

### 4.2 Density estimates

Our SCR models provide the first estimates of density for dingoes in a coastal, peri‐urban ecosystem in South East Australia. On average, our estimates of adult dingo density (0.07/km^2^) are similar to those reported in South East Australia, in Namadgi National Park (0.06/km^2^; Forsyth et al., 2019), the study area of which was of a similar size to ours. However, our model identified strong variation in dingo density within our study region due to gradients of resource supplements. Estimates of density varied spatially across the study area depending on resource supplement, ranging from 0.025/km^2^ in areas without resource supplements, to 0.43/km^2^ in areas with the highest resource supplements (Figure 5a). The lower estimate in more ‘natural’ areas is lower than the reported value from Namadgi National Park (Forsyth et al., 2019), but consistent with the 0.03 dingoes/km^2^ in eastern Victoria (Robley et al., 2018), which was conducted over a much larger area, and likely included areas of both high and low dingo density. Density estimates in all sessions were lower than those reported in the tropics (0.15/km^2^). South‐eastern Australian forested ecosystems may provide fewer food resources for dingoes than are found in tropical northern Australia, even with sources of anthropogenic resource supplements available in our study area. Interestingly, Gabriele‐Rivet et al. (2020) found no impact of an anthropogenic resource supplement on dingo density in their study (Gabriele‐Rivet et al., 2020). Accounting for accessibility of resource supplements is difficult when the presence of this resource can be so stochastic, and the daily energetic requirements of individual dingoes can vary considerably (Tatler et al., 2021). The anthropogenic resource supplement for dingoes in North Australia may not have provided sufficient resources for dingoes to obtain higher densities in this area. Despite the cost and lengthy duration of SCR studies, further dingo density estimates across the continent are needed to compare the impacts of food availability and productivity on dingo density and movement parameters.

## 5 CONCLUSIONS

This study highlights the complexities of population dynamics in heterogeneous environments, and consequently their management, in areas where humans use the land for different purposes such as for permanent habitation, recreation, or conservation. We found that density increased with increasing human population density and that *σ* decreased with increasing human population density. Additionally, we found significant differences in *σ* and *g*_0_ between survey periods, suggesting that dingoes are increasing their home ranges in non‐peak tourism periods, although we cannot discount the possibility that this may be related to species biology rather than the effects of potentially reduced anthropogenic resource supplement in this period. Our results demonstrate that generalist carnivores can attain higher densities and have smaller home ranges in areas with higher levels of human population density than in natural areas, and suggest that resource subsidies may contribute to elevated densities and altered ranging characteristics, both of which are relevant for wildlife management.

## AUTHOR CONTRIBUTIONS

**Brendan F. Alting:** Conceptualization (lead); data curation (lead); formal analysis (lead); investigation (lead); methodology (lead); project administration (equal); writing – original draft (lead); writing – review and editing (lead). **Benjamin J. Pitcher:** Conceptualization (equal); investigation (equal); methodology (equal); supervision (equal); writing – review and editing (equal). **Matthew W. Rees:** Formal analysis (equal); methodology (equal); writing – review and editing (equal). **José R. Ferrer‐Paris:** Formal analysis (equal); methodology (equal); writing – review and editing (equal). **Neil R. Jordan:** Conceptualization (equal); data curation (equal); funding acquisition (equal); investigation (equal); methodology (equal); project administration (equal); supervision (equal); writing – review and editing (equal).

## CONFLICT OF INTEREST STATEMENT

No actual or potential conflicts of interest are declared by the authors.

## Data Availability

All data and code used for analysis is available online: https://github.com/Brendan‐Alting/Carnivore‐Human‐Population‐Density.
